# Undergraduate peer assisted learning tutors’ performance in summative anatomy examinations: a pilot study

**DOI:** 10.5116/ijme.5aa3.e2a6

**Published:** 2018-03-30

**Authors:** Andee Agius, Isabel Stabile

**Affiliations:** 1 Department of Anatomy, Faculty of Medicine and Surgery, University of Malta, Malta

**Keywords:** Peer assisted learning, anatomy, spotting exams, medical education

## Abstract

**Objectives:**

To compare summative anatomy examination results of Peer Assisted Learning (PAL) tutors and learners in the same undergraduate classroom.

**Methods:**

Comparative study of Year-1 medical students who did/did not serve as PAL tutors. PAL tutors gave six hours of teaching in lower limb anatomy. Percent marks for written and spotting examinations were compared between PAL tutors and PAL learners.

**Results:**

The 12 self-selected PAL tutors were not significantly different from their peers (n=191) in terms of age or nationality, but 20% were female compared to 51% of PAL learners. Except for upper limb anatomy, PAL tutors performed at the same level as their tutees in all basic science examinations taken before PAL was introduced. PAL tutors performed better (M=89.0, SD=8.2) in the lower limb examinations than PAL learners (M=79.7, SD=13.0), but these differences were only statistically significantly bigger in the subject they had taught (t_(184)_ = 2.40, p=0.002). Overall PAL tutors performed better in all anatomy spotting exams in both pre-clinical years (Year-1: M=80.4, SD=7.4; Year-2: M=74.8, SD=3.4) compared to PAL learners (Year-1: M=75.1, SD=6.6; Year-2: M=67.2, SD=3.0; (t_(1)_ = 4.2, p=0.07).

**Conclusions:**

Undergraduate PAL tutors performed better than PAL learners in the subject they taught and continued to do so in all anatomy spotting exams, even after the PAL experience had ended, suggesting that actively involving anatomy students as PAL tutors should be encouraged especially among undergraduate medical students.

## Introduction

The concept of Peer Assisted Learning (PAL) in medical education dates back to the first century AD.^1^ Possibly the first published study of the effectiveness of senior clinical students as preceptors for freshman clinical students was that of Resnick and MacDougall,^2^ closely followed by that of Rund et al.^3^ The positive findings of these early studies paved the way for the explosion of PAL in medical education.

Surveys of US medical schools have shown that the vast majority offer some form of PAL during their medical program and approximately one third also provide a formal medical student-as-teachers programme to guide them in their teaching roles.^4^^,^^5^ However, the literature is obscured by the fact that there are at least 15 other widely used synonyms for PAL including collaborative tutoring, cooperative learning, facilitative peer mentoring, supplemental instruction or merely peer/near-peer teaching. Olaussen et al. have recently tried to clarify the nomenclature based on the relationship and ratio between student and teacher.^6^ Interest in PAL in medical, dental, veterinary and allied health courses is increasing worldwide as evidenced in the vast number of published studies regarding its conceptual and curriculum framework, as well as narrative, critical and systematic reviews.^7^^-^^15^ The benefits of PAL are two-fold. In addition to offering an educational exercise to the student tutors and learners, it also addresses the worldwide acute shortage of qualified lecturers, especially important in some pre-clinical disciplines including anatomy and physiology.^8^^,^^16^^,^^17^

Systematic reviews and meta-analyses have shown PAL to be an effective and enriching method for students to learn with knowledge and skills outcomes that are not significantly different than those taught by faculty.^11^^,^^14^ For example, the randomised controlled trial of Haist et al. showed that Year-4 medical students were as effective as faculty in teaching physical examination skills to Year-1 students.^18^ Similar findings were reported by Graham et al. regarding musculoskeletal examination.^19^ The randomised comparison study of Steele et al. showed that there was no objective difference in Year-2 student performance based on whether the PBL facilitator was a faculty member or peer group member.^20^ Thus, although PAL tutors could not possibly have the content knowledge or teaching skills of experienced faculty tutors, these small randomised studies in selected subject areas show that the students achieve comparable results.

The question of whether PAL improves student outcomes in the basic sciences (anatomy and physiology) has been examined using before/after studies. For example, Jackson and Evans^21^ showed improvement in Year-1 physiology exam scores in one theme (blood) in the year PAL was introduced compared to the previous year’s cohort. Otherwise, all exam results were virtually identical. In the study of Nnodim,^22^ 80 Year-2 students performed only half of the trunk dissection, relying on their peers for instruction on the other half. PAL participants performed significantly better in a written and practical exam than the control group who dissected full-time. Similarly, Rengier and colleagues^23^ reported that based on pre/post testing, students enrolled in a three-day peer-led revision course in anatomy in preparation for the German national exam, performed as well as those in two comparison groups. By contrast, Batchelder and colleagues^24^ studied an intensive pre-clinical (non-dissection) teaching programme designed and implemented by senior medical students (including small group teaching with short lectures and case studies) and showed no statistically significant difference in change in rank between those who attended PAL sessions and those who did not.

An adaptation of the concept of PAL is that of Reciprocal Peer Teaching (RPT) wherein students alternate roles as teacher and learner. In apparently the first such published study set in the gross anatomy laboratory, Hendelman and Boss asked 66 Year-1 medical students to compare their experience of RPT with that of staff teaching.^25^ The majority reported that they acquired as much knowledge of anatomy topics taught by their peers and greater knowledge of those topics that they had taught themselves. Similar results regarding perceived retention of anatomical knowledge and communication skills were reported in an anatomy lab setting by Krych and colleagues.^26^

In terms of actual exam results, in the RPT study of Yeager and Young in which dissectors gave a prosection demonstration to the other students assigned to their cadaver, the participants scored better than the national average on the anatomy part of the National Board Examination, in spite of fewer contact hours than the national average.^27^ Bentley and Hill showed there was no significant difference in practical anatomy exam grades between the RPT cohort and previous non-RPT classes in a gross anatomy laboratory setting, suggesting that RPT was not detrimental to anatomy learning.^28^ However, student feedback highlighted two important possible disadvantages: less dissection time (as students would be teaching during half the time) and the possibility that the quality of peer teaching might be unreliable. However, the study of 227 Tanzanian students by Manyama et al. showed that not only did anatomy exam performance significantly improve (by 5%) after RPT in the dissection lab but low performing students prior to RPT benefited most in terms of examination results.^29^ An important bias in this study (pointed out by the authors), was that before the RPT was introduced, students had dissected more difficult regions (including head and neck and neuroanatomy) compared to the abdomen and pelvis during the RPT section of the study.

A further adaptation is the concept of near peer-assisted learning (N-PAL) wherein more senior medical students, some in their clinical years, teach anatomy to their junior colleagues.^30^ The small study of Cantwell et al. showed that supplemental exercises offered by near-peers increased student knowledge and confidence in their dissection techniques.^31^ Duran et al. showed that practical near-peer teaching anatomy sessions (especially dissection) were viewed more favourably compared to the theoretical components in a large cohort with a low faculty to student ratio.^32^ As first suggested by Blanc and Martin, there is now consensus that the N-PAL approach promotes self-directed learning.^33^

A further adaptation is that of Doughnut Rounds (DRs), an innovative approach to self-directed learning first introduced in the context of surgery and orthopaedics.^34^^,^^35^ Zhang and colleagues extended the concept to clinical anatomy in their pilot study of Year-1 medical students who attended six weekly hour-long sessions in small groups.^36^ Each student prepared five questions on a different clinical anatomy topic every week. During each DR session, students took turns to ask their questions to others in the group. Each incorrect/correct answer was then explained to the students by the Year-4 N-PAL facilitator. Not only did the average pre/post MCQ scores increase by almost 40%, but participation in the sessions was reflected in a significant improvement in both written and spotting examination results in those students who were previously behind academically when compared with non-DR participants in the same cohort. The majority of students reported that formulating questions aided in their retention of knowledge and that the sessions were valuable in relation to the time and effort in preparing for them.

Quite apart from gain in content-specific knowledge, students in the role of teachers are more likely to report improved self-esteem, and develop feelings of proficiency, autonomy as well as a positive attitude towards the subject.^8^ This supports the earlier findings of Krych et al. that anatomy RPT stimulates teamwork, decision making, leadership and also assists students in refining their oral presentation skills.^26^ In line with this, Manyama and colleagues also reported improvement in confidence and presentation skills amongst the benefits attributed to RPT in the anatomy dissection lab.^29^ Providing information, serving as role model and facilitator are all transferable skills when near-peer learners and tutors become doctors.^26^^,^^37^^,^^38^ Moreover, Amorosa et al. report that compared to prior to participating in PAL, peer tutors make the connection between teaching and doctoring much more easily.^39^  Moreover, it has been shown that improvement in leadership skills and confidence, as well as an increased ability to provide constructive feedback, were much more significant in PAL tutors than in their tutees.^12^^,^^40^ Beyond teaching per se, using case studies, Furmedge and colleagues showed other domains where PAL can benefit students including resource development, peer-assessment, education research, and evaluation as well as mentoring and support.^41^

A number of small studies have reported the impact of PAL on the exam performance of the tutors themselves, e.g., in ACLS and EKG interpretation, musculoskeletal examination and ultrasound, GI/Haematology, pharmacology and patient-centered interviewing skills.^42^^-^^47^ Apart from the latter, all studies showed statistically significant short-term knowledge acquisition among PAL tutors. In the prospective randomised intervention study of Gregory et al. the significant learning gains were still present when subjects were re-tested 60 days later.^42^ To date, no studies have detected significant harm to PAL tutors as a result of participation in this activity.

By contrast, very few studies have examined the effects of the PAL process on the exam scores of the PAL tutors themselves in the basic science disciplines. In one such study, Wong and colleagues matched 199 supplemental instruction leaders (upper-level PAL tutors between 1996 and 2001) for age, gender, and admission characteristics with controls (non-teachers) and demonstrated that the former had significantly higher USMLE Step 1 and 2 scores as well as final medical school GPA.^48^ In this study, the leaders provided intensive small group teaching (3 hours weekly for one semester) and had themselves received extensive training as well as regular feedback from the course coordinators. However, it should be emphasised that medical students in the US are graduates, and hence older, and some may even have previous teaching experience. Moreover, this study did not specify which of the basic science disciplines were taught by the supplemental instructors.

This study aimed to compare the summative anatomy examination results of PAL tutors with those of PAL learners in the same undergraduate classroom during the first two years of medical school. We hypothesized that PAL tutors would perform better than PAL learners only in the subject they had taught.

## Methods

### Context

The Faculty of Medicine at the University of Malta recently introduced a modular ECTS-based system throughout its course. The course is structured as a traditional basic science pre-clinical component (2 years) followed by a clinical component (3 years). Within the pre-clinical course, the teaching and assessment for each study unit is primarily system-based, such that within the Respiratory, Renal, Cardiovascular, Gastrointestinal and Reproductive systems, all physiology and anatomy is examined together at the end of the respective semester.

### The exams

Each study unit is assessed via a written examination at the end of the semester. In addition, the anatomy component of each study unit is assessed through a spotting test which carries a small (10-20%) portion of the final overall marks. There are separate study units for Cell Biology and Biochemistry (Semester 1) and Molecular Biology and Genetics (Semester 2) that contain no anatomy. The wholly anatomy-based Musculoskeletal system is taught and examined in two parts: Upper Limb in semester 1 and Lower Limb in semester 2 of the first year. Each 2-hour-long written examination is composed of 100 Best of Five questions, most of which test detailed knowledge of anatomy and physiology with little emphasis on clinical relevance. The spotting anatomy examination consists of 25 labeled images of prosections to be identified each semester. The University of Malta does not use standard setting in any of its examinations. All questions are prepared by the examiners (who are also the tutors). The exam paper is then reviewed by a separate examiner whose feedback as to the suitability of the questions is incorporated into the final exam paper.

### Study design and participants

The study took the form of a comparative study of students who did/did not serve as PAL tutors. During the first semester, all Year-1 students took the same modules (Upper Limb, Respiratory System, Cell Biology and Biochemistry, Organisation of the Body and Ethics) and no PAL was conducted. At the start of the second semester, all Year-1 students were given the first-ever opportunity to participate in PAL in our curriculum. Twelve volunteers were selected by ballot to participate as PAL tutors. The supervising faculty member encouraged PAL tutors to familiarise themselves with the anatomy of the leg and ankle and practice teaching this to each other. Mid-way through the semester, each PAL tutor gave two mini-lectures and four dissection sessions to the rest of the class. During these sessions, the PAL tutors demonstrated the relevant anatomy on prosections using a video-dissection format to the rest of the class. The theoretical and practical PAL teaching sessions were supervised at all times. Following completion of the module, all the students underwent the same high stakes examinations in all modules. As the study involved only anonymized data, no ethical issues were noted. The study was approved by the University of Malta Ethics Review Board.

### Data collection

Age, gender and nationality were extracted from class registers. The Year-1 and 2 final examination results of the PAL tutors and PAL learners were extracted from the Student Information Management System and anonymised using unique identifiers. In order to prevent double-counting, only the first-sit exam marks were included.  The percent marks for the written and practical components of each study unit were compared between PAL tutor and PAL learner groups.

### Statistical analysis

All marks were exported from Excel to SPSS Version 21.0 (IBM Corp., Armonk, NY, USA). Descriptive statistics were used to analyse the data. Continuous variables were reported as means with standard deviation (M and SD). The t-test was utilized to compare means, with significance defined when p < 0.05.

## Results

A total of 191 Year-1 students sat for the examinations. The 12 PAL tutors were not significantly different from their peers in terms of age or nationality. However, only 20% of PAL tutors were female compared to 51% of PAL learners.

All marks were reported out of 100. In the semester prior to their participation in PAL, PAL tutors only performed statistically significantly better (M=70.5, SD=7.4) than PAL learners (M=61.8, SD=11.8) in the Upper Limb written anatomy examination (t_(__184)_=2.51, p=0.002).

PAL tutors performed better (M=89.0, SD=8.2) in the lower limb examinations than PAL learners (M=79.7, SD=13.0), but these differences were only statistically significantly better in the practical anatomy spotting test of the lower limb i.e., the subject they had taught (t_(__184)_=2.40, p=0.002) Table 1.

Overall, PAL tutors performed significantly better in the lower limb spotting exam (M=89.0, SD=8.2) compared to all other anatomy spotting exams (M=81.5, SD=3.7) they sat for in the same academic year (t_(__60)_ =2.50, p=0.01).

In Year-2, PAL tutors also scored significantly higher marks (M=78, SD=10.3) in the Year-2 spotting (but not written) examination of the Anatomy of the Gastrointestinal Tract than PAL learners (M=70.6, SD=17.6; t_(__12)_=2.88, p=0.04). They also scored significantly higher (M=70.8, SD=8.0) than PAL learners (M=64.9, SD=9.4) in the Integrated Biomedical Sciences study unit (t_(__12)_=2.62, p=0.04), which is a summative exam over four semesters of basic science learning. Their marks in all other modules were not significantly different.

Overall PAL tutors performed better in all anatomy spotting exams in both pre-clinical years (Year-1: M=80.4, SD=7.4; Year-2: M=74.8, SD=3.4) compared to PAL learners (Year-1: M=75.1, SD= 6.6; Year-2: M=67.2, SD=3.0; (t_(1)_ = 4.2, p=0.07).

## Discussion

There is some evidence that PAL in the gross anatomy laboratory benefits PAL tutors themselves, in terms of global exam results.^27^^,^^29^^,^^48^ This pilot study adds to this body of knowledge by showing similar results for six hours of PAL tuition in the form of mini-lectures and structured dissection sessions on individual written and practical anatomy study units over the first two years of pre-clinical studies.

PAL tutors are a self-selected group of often high academic achievers, a factor which has been shown to relate to future academic success.^49^^,^^50^ However, this alone cannot explain our findings, as the PAL tutor volunteers performed at the same level as their tutees in almost all basic science module examinations taken before PAL was introduced. The only anatomy module exception was in the upper limb where tutors scored significantly better in both the written and practical spotting tests. Moreover, the PAL tutors did not improve their written or practical scores in the subject they taught (lower limb) compared with their results for the upper limb in the previous semester. Thus, it is unlikely that those who volunteered for PAL were overall academically stronger than their peers to start off with. Instead, we believe that those who volunteered, did so because they had enjoyed and performed well in the upper limb examination in the previous semester. However, given that female students tend to obtain higher marks, the preponderance of females among tutees may have biased the results by diluting the possible effect of PAL on the tutors’ exam performance.

**Table 1:** Comparison between marks obtained in upper limb (before PAL) and lower limb (after PAL)

Statistical Measures	Upper Limb (Semester 1: Before PAL)				Lower Limb (Semester 2: After PAL)			
	Written		Practical		Written		Practical	
	PAL Tutor	PAL Learner	PAL Tutor	PAL Learner	PAL Tutor	PAL Learner	PAL Tutor	PAL Learner
Mean	70.5%	61.8%	84.8%	77%	71.8%	65.2%	89%	79.7%
SD	7.4	11.8	11.5	14.6	10.2	11.3	8.2	13.0
% Difference	8.7		7.7		6.5		9.3	
p value	0.002		0.045		0.053		0.002	

There is some evidence that both preparing to teach and the process of teaching itself result in greater knowledge gains compared to studying alone.^42^^,^^51^^,^^52^ Given that students who prepare to teach spend more time on the subject, we were surprised to find that in this study, teaching preparation did not improve the written or practical scores in the subject they taught (Lower Limb) compared with the most similar content module in the previous semester (Upper Limb). Thus, although the PAL tutors were a tiny, self-selected and motivated group, the process of preparing to and then actually teaching did not appear to affect retrieval of anatomical knowledge in a similar domain (Lower versus Upper Limb). Nevertheless, as expected, PAL tutors performed better than their tutees in the subject they taught, but surprisingly only in the spotting anatomy exam. One would have expected that their additional preparation time would have been reflected in higher written scores too. We believe that this is probably because each student only taught for six hours and the teaching was limited to the leg and ankle, which is just a subset of the whole lower limb curriculum. It is also possible that the PAL tutors felt confident that preparing themselves to teach these topics was sufficient exam preparation and hence chose to focus their studies on the examinations of the other four concurrent modules that semester.

The PAL tutors had several additional hours of exposure to the material they were teaching (through personal preparation to become PAL tutors), but they had no equivalent exposure to the remaining anatomy content. Nevertheless, when all practical anatomy spotting exams in the pre-clinical years are taken into account, the average overall score of PAL tutors was higher than that of PAL learners. It is possible that the PAL tutors were academically stronger than their peers, and/or preferred learning anatomy over the other basic sciences, but it is also conceivable that through their preparation to teach, our PAL tutors learned to reflect on their knowledge gaps which may have led them to utilise a deeper learning approach, resulting in improved performance in other (more difficult) anatomical topics.^53^^,^^54^ It is also feasible that the enthusiasm of the faculty member facilitating this first-ever PAL activity in our curriculum may have ignited the PAL tutors’ interest in anatomy, motivating them to work extra-hard to achieve better results themselves. Given that these were Year-1 medical students and that this was their very first opportunity to participate in PAL, it is also possible that the tutors who volunteered did so because they had previous teaching/tutoring experience (which they had enjoyed). Any of these could explain why PAL tutors continued to perform better in all anatomy spotting exams than their peers, even after their very brief PAL experience.

Iwata and colleagues showed that timing influences the link between tutoring and exam performance, as those who tutored closer to examinations performed slightly better.^55^ However, our findings cannot be explained on this basis as all students sat for final exams approximately two months after the PAL sessions had ended.

Adequate training and continuous faculty supervision are considered important for PAL to be successful.^40^ The focus group interviews of Alvarez et al. showed that a preparatory training course for PAL tutors that encompasses dealing with students in difficulty, handling group dynamics, keeping students motivated, and time management is ideal.^56^ In our study, the PAL tutors received no formal training, but were continuously supervised and supported by the faculty coordinator.

We should also point out that the majority of the PAL tutors and learners in this study joined the medical course after 13 years of school education. Lacking prior undergraduate education means that our PAL learners may not yet have learned how to learn. This group of “green learners” are ripe for exposure to a PAL, as they have the most to gain from experience. The findings of this pilot study would suggest that all students should be given the opportunity to teach anatomy to their peers, as has indeed been widely recommended.

### Limitations

The sample size in this pilot study was limited by the number of sessions to be taught and the class size. Moreover, the relatively small number of PAL tutors in this study were a self-selected group of highly motivated volunteers who knew they were participating in a study. It is also possible that the hours they spent preparing for the sessions may have positively influenced their grades.  The low ratio of female compared to male PAL tutors may also have negatively impacted the results, as in general, female students perform better in summative examinations. All medical students at the University of Malta have achieved a very high grade in Chemistry and Biology (approximately equivalent to A* in the UK A level system). Notwithstanding, it is possible that some other variable that encouraged student participation in the PAL programme may somehow independently have affected their academic performance. Characteristics such as age, ethnicity, previous formal teaching experience were not taken into account. Moreover, we used data from only one academic year group. Ideally, the study would be repeated across several year-groups and with a larger sample size.

## Conclusions

This pilot study aimed to compare the anatomy final examination results of PAL tutors with those of PAL learners in the same undergraduate classroom during the first two years of medical school. We expected PAL tutors to perform better in the subject they taught (lower limb), but their marks were only significantly higher in the practical spotting examination. The PAL tutors were self-selected, but they did not appear to be better exam takers than the PAL learners, as they performed equally in most written basic science exams taken before or after the PAL sessions took place. However, PAL tutors continued to perform better in all anatomy spotting exams than their peers, even after the PAL experience had ended. Although PAL is a reciprocal process, in this pilot study, it appeared to benefit participating anatomy tutors more than learners. The implications for medical education is to encourage medical educators to involve anatomy students actively as PAL tutors, especially among undergraduate medical students.

### Conflict of Interest

The authors declare that they have no conflict of interest.
